# Supplementary material for: Epidermal progenitors suppress GRHL3-mediated differentiation through intronic polyadenylation promoted by CPSF-HNRNPA3 collaboration
Source: Nat Commun. 2021 Jan 19;12:448. doi: 10.1038/s41467-020-20674-3 (PMC7815847; doi:10.1038/s41467-020-20674-3)
Supplement: Supplementary file 1 — Supplementary Information [file 41467_2020_20674_MOESM1_ESM.pdf]

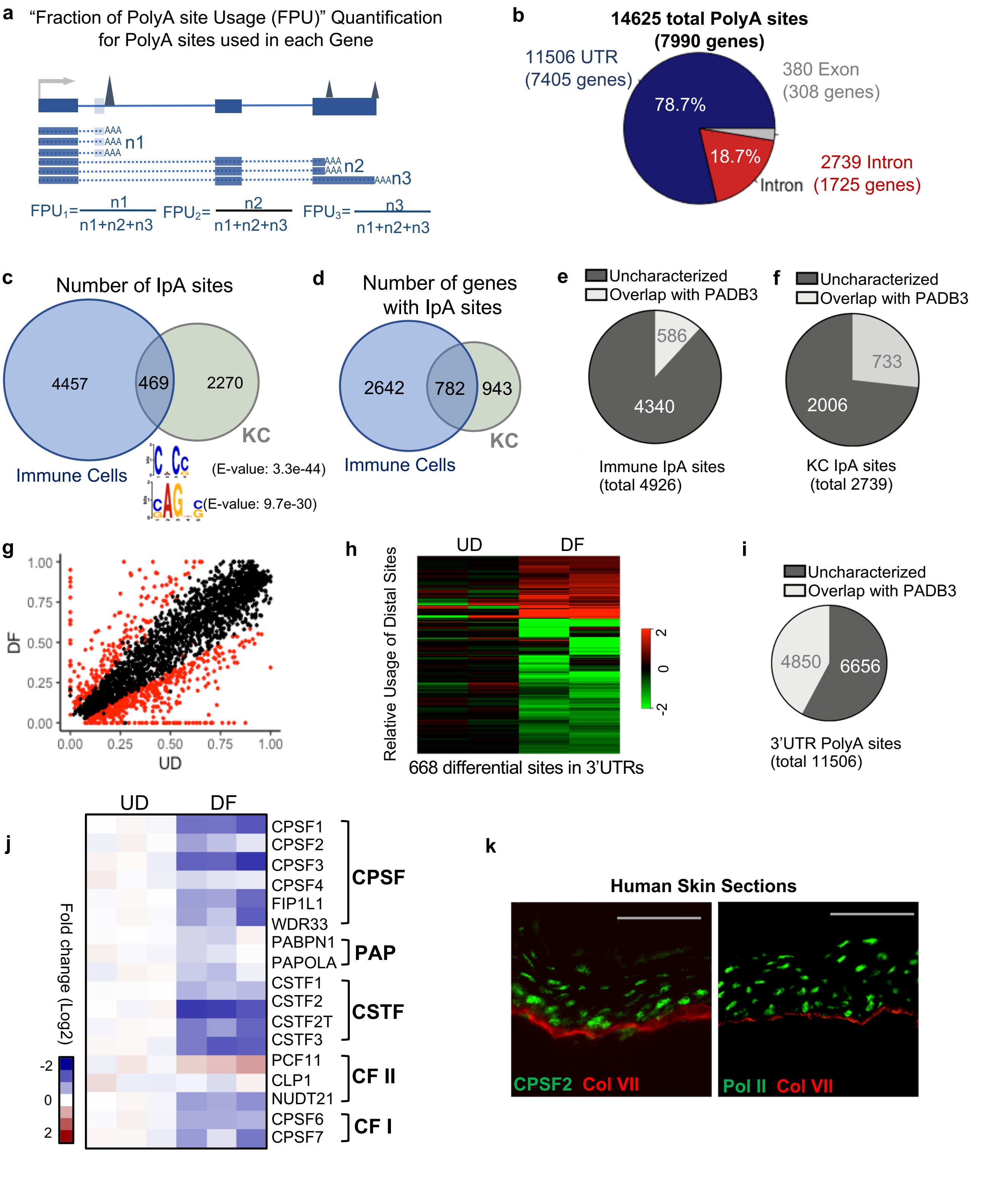

Supplementary Figure 1. Keratinocyte Differentiation Involves Altered PolyA Usage and CPSF Downregulation. (Legend included on the next page.)

Supplementary Figure 1. Keratinocyte Differentiation Involves Altered PolyA Usage and CPSF Downregulation. (a) Illustration showing calculation of the "Fraction of PolyA Usage (FPU)" for PolyA sites in each gene. All PolyA sites are assigned to genes based on their genomic coordinates. FPU for a specific PolyA site is calculated as counts of this site divided by counts of all PolyA sites associated with this gene. (b) Pie chart showing the distribution of the 14625 total PolyA sites identified from our 3'READS+ experiment. (c) Venn diagram comparing the Intronic polyadenylation (IpA) sites identified in keratinocytes versus the IpA sites identified from the Immune system[1]. Top motifs associated with the IpA sites in keratinocytes, as compared to the IpA sites associated with the immune cells, are included below. (d) Venn diagram showing the genes associated with IpA sites in immune cells and in keratinocytes. 62% of genes associated with IpA sites in keratinocytes overlap with the genes associated with IpA in immune cells. (e,f) Pie chart showing the overlap of IpA sites with the PADB3 database[2]. (g) Scatter plot showing the distribution of Distal Site Usage within the 3' untranslated regions (3'UTRs), among the 2727 genes with multiple PolyA sites in 3'UTRs, comparing undifferentiated and differentiated keratinocytes. 668 distal 3'UTRs that are differentially used (fold change >1.5) are highlighted in red. (h) Heatmap showing the fold change of Distal Site Usage between UD vs DF, among the 668 differentially used 3'UTR PolyA sites. (i) Pie chart showing the overlap of 3'UTR PolyA sites with the PADB3 database. (j) Heatmap comparing the relative expression of genes encoding complexes involved in polyadenylation, including CPSF, PAP, CSTF, CF I and CF II, based on our RNA-seq data comparing undifferentiated (UD) and differentiated (DF) keratinocytes (n=3). (k) Immunostaining of CPSF2 or Pan- Pol II in human skin section. (CPSF2 or Pan- Pol II = green; collagen VII basement membrane marker = red. Scale bar = 50uM. Representative images of 17 CPSF images from 3 independent experiments and 6 images of Pol II staining are shown). Source data are provided as a Source Data file.

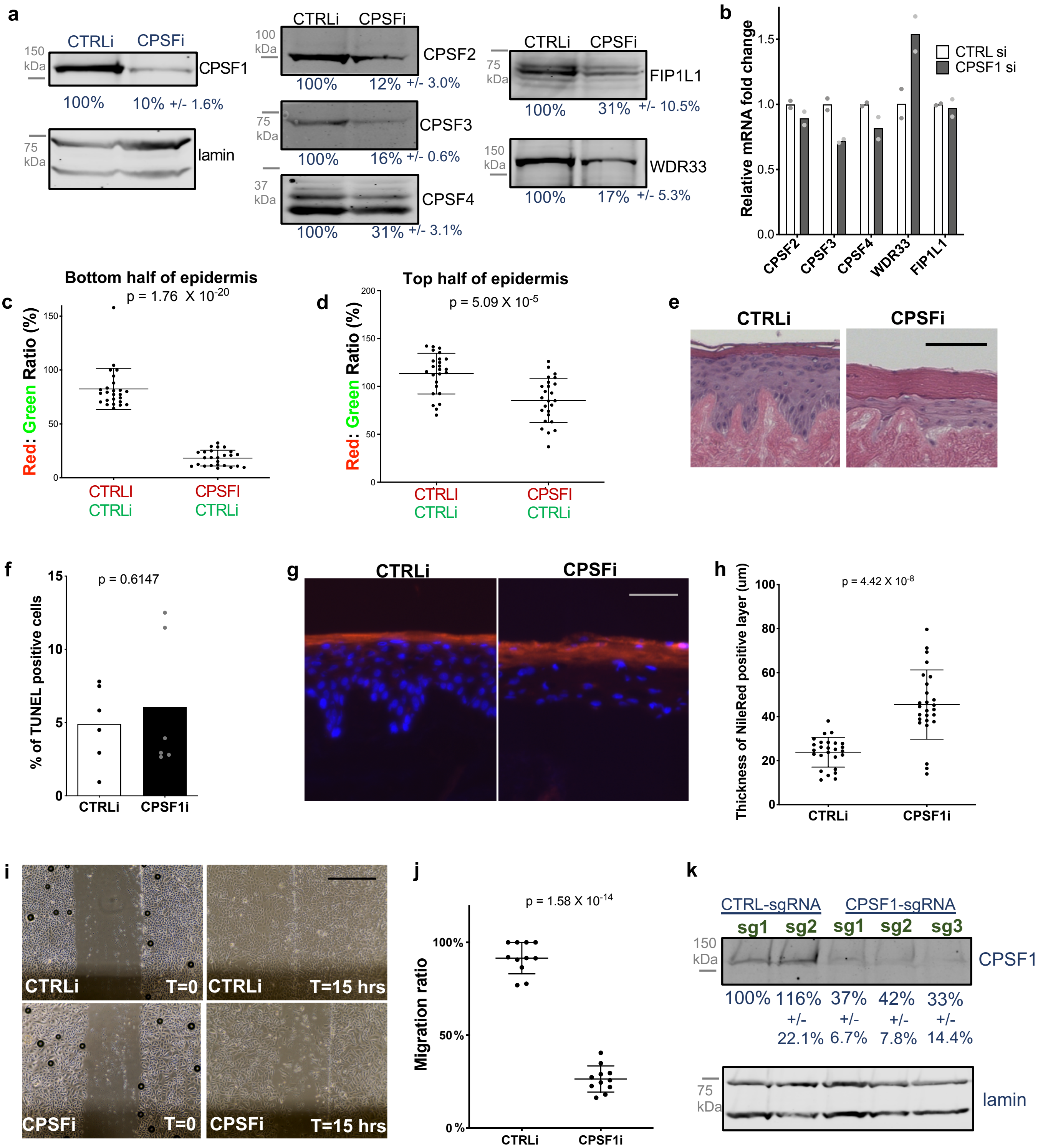

Supplementary Figure 2. CPSF Downregulation Impairs Keratinocyte Regeneration and Migration. (Legend included on the next page.)

Supplementary Figure 2. CPSF Downregulation Impairs Keratinocyte Regeneration and Migration. (a,b) Western blotting detecting the protein levels of CPSF subunits, comparing keratinocytes nucleofected with CPSF1 siRNA vs control siRNA. Quantification of relative expression with 3 replicates is indicate below each blot. (b) qRT-PCR showing the influence of CPSF1 knockdown on the expression of other CPSF subunits at the mRNA level. Dots represent data points from technical replicates. (c,d) Quantification of red:green ratio comparing the bottom or top half of regenerated epidermal tissue with CTRLi/CTRLi versus CTRLi/CPSFi keratinocytes (n=25 images per condition, t-test, two-tailed, error bars are represented as mean values +/- SEM). (e) H&E staining of organotypic epidermal tissues using keratinocytes with CPSFi vs. CTRLi (Scale bar = 100um). Representative image from 5 images of 2 biological replicates are shown. (f) Quantification of TUNEL assay (n=6 images per condition) comparing epidermal tissue regenerated by keratinocytes nucleofected with CPSF1 siRNA vs control siRNA (p=0.6147, t-test, two-tailed). (g) Representative images of Nile Red staining comparing regenerated epidermal tissue with CPSF knockdown vs control, from 26 images of 2 biological replicates. (h) Quantification of the thickness of Nile-red positive regions (n=26 images per condition, Scale bar = 500um, t-test, two-tailed, error bars are represented as mean values +/- SEM). (i,j) Comparison of keratinocyte migration in scratch assay between keratinocytes nucleofected with CPSF1 siRNA vs control siRNA, with representative images and quantification (n=11 images per condition, Scale bar = 500um, t-test, two-tailed, error bars are represented as mean values +/- SEM). (k) Western blotting showing the efficiency of CRISPRi in suppressing CPSF1 gene expression, comparing 3 sgRNAs targeting CPSF1 and 2 control sgRNAs. Quantification of 2 replicates is indicate below. Source data are provided as a Source Data file.

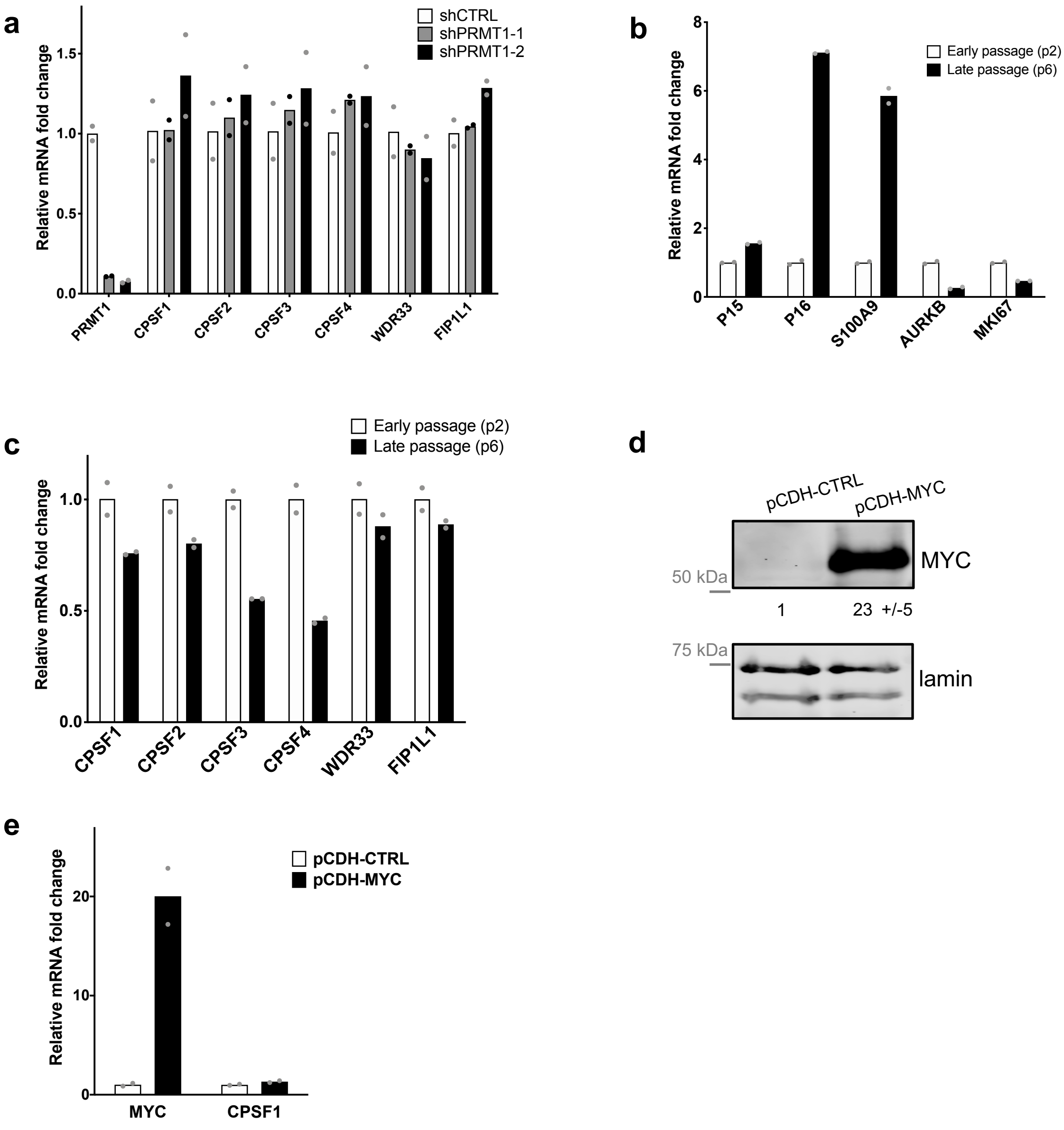

Supplementary Figure 3. Regulation of CPSF1 Expression in Keratinocytes. (a) qRT-PCR quantification of CPSF subunit mRNA expression, comparing keratinocytes with PRMT1 knockdown versus control. Dots represent data points from technical replicates. (b,c) qRT-PCR quantification of differentiation marker genes, proliferation marker genes, and CPSF subunit genes, between early-passage keratinocytes (p2) and late-passage keratinocytes (p6). Late passage keratinocytes were cultured 20 additional days as compared to early-passage keratinocytes. Dots represent data points from technical replicates. (d) Western blotting showing MYC overexpression versus vector control in differentiated keratinocytes. Quantification of fold change is indicate below (n=2). (e) qRT-PCR quantification of MYC and CPSF1 mRNA expression, comparing keratinocytes infected with MYC overexpression vector versus control vector. Dots represent data points from technical replicates. Source data are provided as a Source Data file.

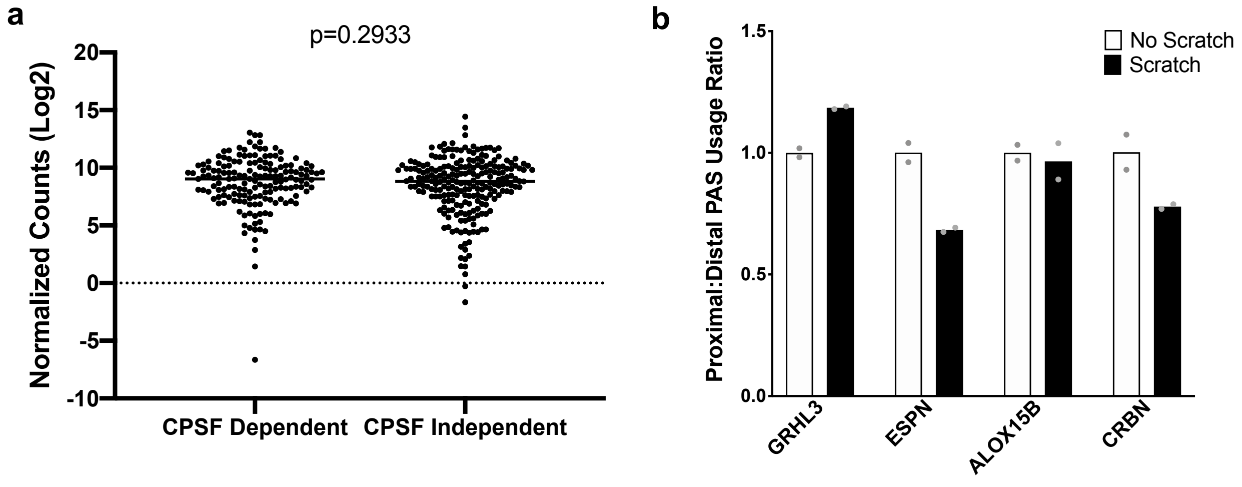

Supplementary Figure 4. Regulation of the Differential IpA Sites in Keratinocytes. (a) Dot-plots showing the expression of genes associated with CPSF-dependent IpA sites (178 sites associated with 165 genes) versus CPSF-independent IpA sites (250 sites associated with 227 genes). Gene expression is represented by normalized counts from DEseq2 output of UD DF RNA-seq libraries. Y-axis shows Log2 transformed average counts of each gene across three replicates. (P=0.2933, two tailed t-test). (b) qRT-PCR quantification of IpA usage between migrating keratinocytes versus control. The migrating keratinocytes were generated by creating a grid of scratches on confluent plates, as compared to control keratinocytes growing on confluent plates without scratches. Dots represent data points from technical replicates. Source data are provided as a Source Data file.

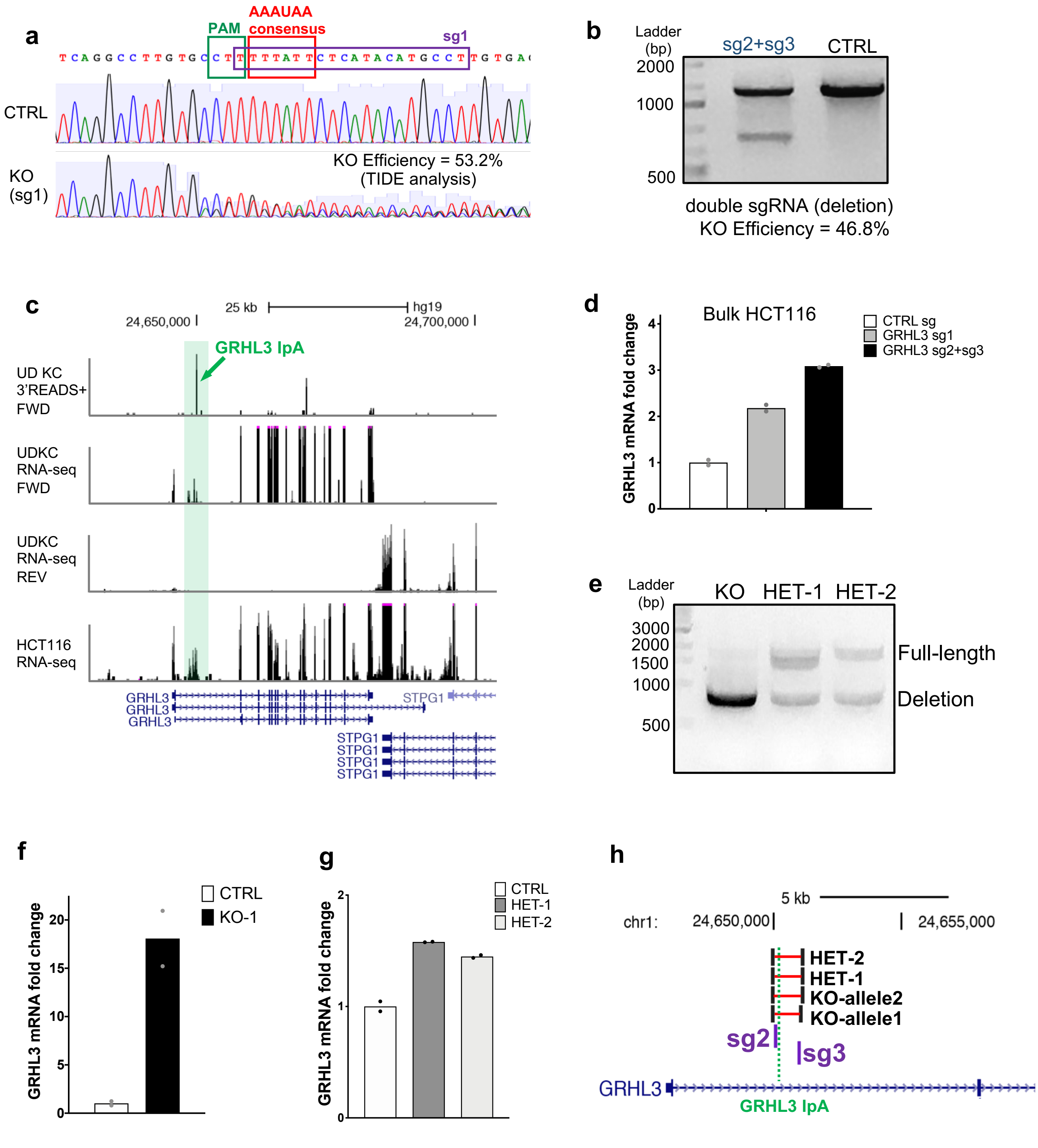

Supplementary Figure 5. GRHL3 IpA Suppresses Its Full-length mRNA Expression. (Legend included on the next page.)

Supplementary Figure 5. GRHL3 IpA Suppresses Its Full-length mRNA Expression. (a) Sanger sequencing results comparing the PCR products of sg1 knockout versus control in keratinocytes. The locations of AAUAAA consensus sequence, PAM, and sg1 are indicated. Based on TIDE analysis, the knockout efficiency is 53.2%. (b) Gel image of the PCR products comparing sg2+sg3 deletion and control in keratinocytes. The calculated knockout efficiency is 46.8%. (c) Genome browser tracks showing that HCT116 cells also have RNA-seq reads near the GRHL3 IpA site, similar to keratinocytes. The HCT116 RNA-seq data were generated by the ENCODE project (GSE33480). Strand-specific RNA-seq tracks from our keratinocyte data are included to clarify the reads associated with GRHL3 versus STPG1 on the other strand. 3'READS+ data track is included to indicate the location of the GRHL3 IpA site. (d) qRT-PCR quantification of GRHL3 full-length mRNA expression, comparing bulk HCT116 cells expressing Cas9 and sgRNAs targeting GRHL3 IpA site versus control sgRNA. Dots represent data points from technical replicates. (e) Gel image showing PCR products corresponding to full-length and deletion of GRHL3 IpA site, using genomic DNA extracted from HCT116 clones with 1 (HET) or both alleles (KO) deleting GRHL3 IpA site. Results from 2 out of 9 HET clones are included as examples. (f, g) qRT-PCR quantification of GRHL3 full-length mRNA expression, comparing KO and HET HCT116 cells versus control. Dots represent data points from technical replicates. (h) Genome browser tracks showing the location of deletions from HET and KO cells, based on BLAT analysis of Sanger sequencing results. The aligned regions are indicated in black, and the deleted regions are indicated in red. Locations of sg2, sg3, and the GRHL3 IpA site are also indicated. Source data are provided as a Source Data file.

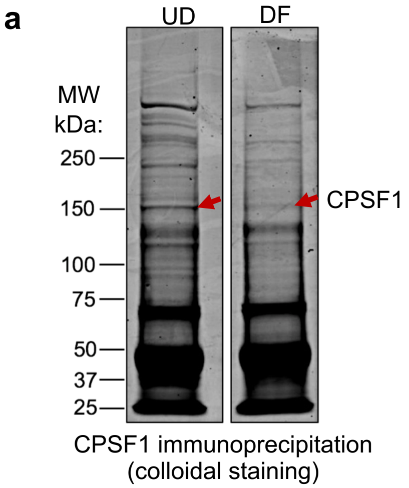

b

	UD	DF
CPSF1	112	69
CPSF2	83	29
CPSF3	20	11
HNRNPA3	9	0
HNRNPUL1	9	0
TAF15	4	0
RBM14	14	0
ILF2	11	0
ELAVL1	19	0
FUS	6	0

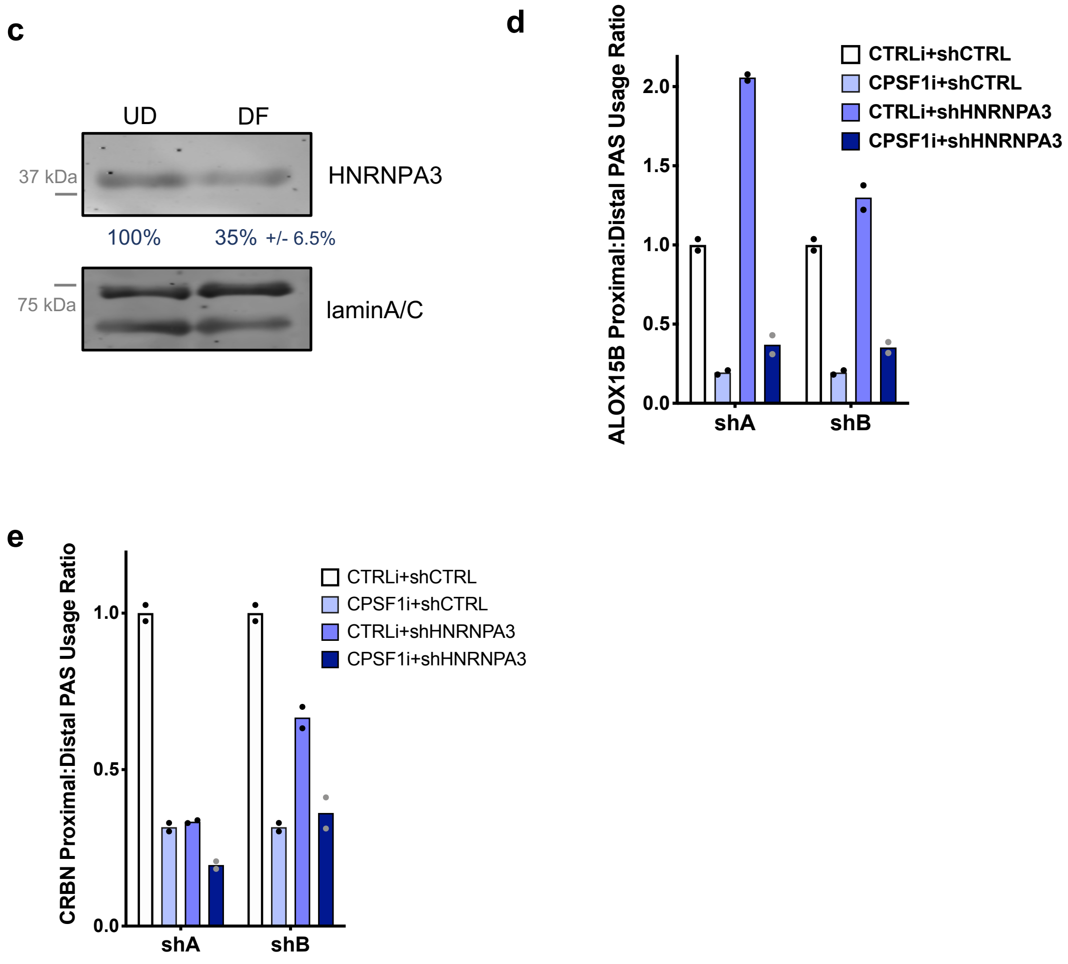

Supplementary Figure 6. Targeted Screen for Factors Enhancing CPSF's Selection of GRHL3 IpA. (Legend included on the next page.)

Supplementary Figure 6. Targeted Screen for Factors Enhancing CPSF's Selection of GRHL3 IpA. (a) Colloidal Staining showing the bands of the co-purified proteins from CPSF1 immunoprecipitation, using undifferentiated (UD) or differentiated (DF) keratinocyte nuclear extraction. Representative image from two biological replicates is shown. (b) Counts of CPSF subunits and RNA-binding proteins identified from our pilot mass-spectrometry experiments, comparing CPSF1 immunoprecipitation between undifferentiated versus differentiated keratinocyte lysate. (c) Western blotting detecting the expression of HNRNPA3 in undifferentiated and differentiated keratinocytes. Quantification of relative HNRNPA3 is indicated below (n=2). (d, e) qRT-PCR quantification of ALOX15B and CRBN IpA usage, between double and single knockdowns of CPSF1 and HNRNPA3. Dots represent data points from technical replicates. Source data are provided as a Source Data file.

Supplementary References

1. Singh, I. *et al.* Widespread intronic polyadenylation diversifies immune cell transcriptomes. *Nat. Commun.* **9**, 1716 (2018).
2. Wang, R., Nambiar, R., Zheng, D. & Tian, B. PolyA_DB 3 catalogs cleavage and polyadenylation sites identified by deep sequencing in multiple genomes. Nucleic Acids Res. 46, D315–D319 (2018).
