# Supplementary material for: Epidermal progenitors suppress GRHL3-mediated differentiation through intronic polyadenylation promoted by CPSF-HNRNPA3 collaboration
Source: Nat Commun. 2021 Jan 19;12:448. doi: 10.1038/s41467-020-20674-3 (PMC7815847; doi:10.1038/s41467-020-20674-3)
Supplement: Supplementary file 3 — Reporting Summary [file 41467_2020_20674_MOESM3_ESM.pdf]

nature research

Corresponding author(s): Xiaomin Bao

Last updated by author(s): Jul 29, 2020

# Reporting Summary

Nature Research wishes to improve the reproducibility of the work that we publish. This form provides structure for consistency and transparency in reporting. For further information on Nature Research policies, see our Editorial Policies and the Editorial Policy Checklist.

## Statistics

For all statistical analyses, confirm that the following items are present in the figure legend, table legend, main text, or Methods section.

| n/a | Confirmed | |
|---|---|---|
| ☐ | ☒ | The exact sample size ($n$) for each experimental group/condition, given as a discrete number and unit of measurement |
| ☐ | ☒ | A statement on whether measurements were taken from distinct samples or whether the same sample was measured repeatedly |
| ☐ | ☒ | The statistical test(s) used AND whether they are one- or two-sided<br>*Only common tests should be described solely by name; describe more complex techniques in the Methods section.* |
| ☐ | ☒ | A description of all covariates tested |
| ☐ | ☒ | A description of any assumptions or corrections, such as tests of normality and adjustment for multiple comparisons |
| ☐ | ☒ | A full description of the statistical parameters including central tendency (e.g. means) or other basic estimates (e.g. regression coefficient) AND variation (e.g. standard deviation) or associated estimates of uncertainty (e.g. confidence intervals) |
| ☐ | ☒ | For null hypothesis testing, the test statistic (e.g. $F$, $t$, $r$) with confidence intervals, effect sizes, degrees of freedom and $P$ value noted<br>*Give P values as exact values whenever suitable.* |
| ☒ | ☐ | For Bayesian analysis, information on the choice of priors and Markov chain Monte Carlo settings |
| ☒ | ☐ | For hierarchical and complex designs, identification of the appropriate level for tests and full reporting of outcomes |
| ☒ | ☐ | Estimates of effect sizes (e.g. Cohen's $d$, Pearson's $r$), indicating how they were calculated |

*Our web collection on statistics for biologists contains articles on many of the points above.*

## Software and code

Policy information about availability of computer code

| | |
|---|---|
| Data collection | Immunoblot : Image Studio Software version 5.2 (LI-COR)<br>qPCR analysis: QuantStudio Design & Analysis Software 1.3.1 (Thermo Fisher)<br>Immunofluorescence: EVOS FL Auto 2 Imaging System Software Revision 2.0.1732.0 (Thermo Fisher)<br>For RNA-seq and 3'READS+, the software and code are described in full in the material and methods section. |
| Data analysis | Immunoblot analysis: Image Studio Software version 5.2 (LI-COR)<br>qPCR analysis: GraphPad Prism version 7<br>Microscopy image processing: ImageJ (NIH)<br>For RNA-seq and 3'READS+, the software and code are described in full in the material and methods section. |

For manuscripts utilizing custom algorithms or software that are central to the research but not yet described in published literature, software must be made available to editors and reviewers. We strongly encourage code deposition in a community repository (e.g. GitHub). See the Nature Research guidelines for submitting code & software for further information.

## Data

Policy information about availability of data

All manuscripts must include a data availability statement. This statement should provide the following information, where applicable:
- Accession codes, unique identifiers, or web links for publicly available datasets
- A list of figures that have associated raw data
- A description of any restrictions on data availability

All data are available either in supplementary data or through accession codes linked in the manuscript.

# Field-specific reporting

Please select the one below that is the best fit for your research. If you are not sure, read the appropriate sections before making your selection.

☒ Life sciences ☐ Behavioural & social sciences ☐ Ecological, evolutionary & environmental sciences

For a reference copy of the document with all sections, see nature.com/documents/nr-reporting-summary-flat.pdf

# Life sciences study design

All studies must disclose on these points even when the disclosure is negative.

| | |
|---|---|
| Sample size | All sample sizes for experiments were determined prior to performing the experiment based on precedent of previous similar experiments using comparable methods. |
| Data exclusions | No data were excluded from the analyses. |
| Replication | All experiments were performed in either technical or biological replicates. All replicates were used in the preparation of each data figure. |
| Randomization | NA. |
| Blinding | NA. |

# Reporting for specific materials, systems and methods

We require information from authors about some types of materials, experimental systems and methods used in many studies. Here, indicate whether each material, system or method listed is relevant to your study. If you are not sure if a list item applies to your research, read the appropriate section before selecting a response.

## Materials & experimental systems

| n/a | Involved in the study | |
|---|---|---|
| ☐ | ☒ | Antibodies |
| ☐ | ☒ | Eukaryotic cell lines |
| ☒ | ☐ | Palaeontology and archaeology |
| ☒ | ☐ | Animals and other organisms |
| ☒ | ☐ | Human research participants |
| ☒ | ☐ | Clinical data |
| ☒ | ☐ | Dual use research of concern |

## Methods

| n/a | Involved in the study | |
|---|---|---|
| ☒ | ☐ | ChIP-seq |
| ☒ | ☐ | Flow cytometry |
| ☒ | ☐ | MRI-based neuroimaging |

## Antibodies

| | |
|---|---|
| Antibodies used | Antibodies from commercial sources:<br>Rabbit anti-CPSF1 Bethyl Cat# A301-580A-T<br>Mouse anti-CPSF1 Santa Cruz Cat# sc-166282<br>Rabbit anti-CPSF2 Bethyl Cat# A301-581A-M<br>Mouse anti-CPSF2 Santa Cruz Cat# sc-165983<br>Rabbit anti-CPSF3 Bethyl Cat# A301-090A-T<br>Mouse anti-CPSF4 Santa Cruz Cat# sc-393316<br>Rabbit anti-WDR33 ABclonal Cat# A9531<br>Mouse anti-FIP1L1 Santa Cruz Cat# sc-398392<br>Mouse anti-Lamin A/C Santa Cruz Cat# sc-376248<br>Mouse anti-Collagen VII Santa Cruz Cat# sc-33710<br>Rabbit anti-GRHL3 Sigma-Aldrich Cat# HPA059960<br>Rabbit anti-Myc Cell Signaling Cat# 5605<br>Rabbit anti-HNRNPA3 Bethyl Cat# A305-815A-T |
| Validation | Myc and HNRNPA3 antibodies were validated by testing on immunoblots with lysates from respective knock-down keratinocytes.<br>GRHL3 antibody was validated by testing on immunoblots with lysates from undifferentiated and differentiated keratinocytes.<br>Validation of all other antibodies purchased from commercial vendors is available on the manufactures' websites. |

# Eukaryotic cell lines

Policy information about cell lines

| | |
|---|---|
| Cell line source(s) | HCT116 |
| Authentication | authentication performed by Northwestern NUseq core facility |
| Mycoplasma contamination | All cell lines and primary human keratinocytes were tested negative, using "MycoFluor Mycoplasma Detection Kit" (cat#M7006) from ThermoFisher |
| Commonly misidentified lines (See ICLAC register) | No commonly misidentified cell lines were used. |
