# Supplementary material for: Epidermal progenitors suppress GRHL3-mediated differentiation through intronic polyadenylation promoted by CPSF-HNRNPA3 collaboration
Source: Nat Commun. 2021 Jan 19;12:448. doi: 10.1038/s41467-020-20674-3 (PMC7815847; doi:10.1038/s41467-020-20674-3)
Supplement: Supplementary file 4 — Description of Additional Supplementary Files [file 41467_2020_20674_MOESM4_ESM.pdf]

Description of Additional Supplementary Files

Supplementary Data 1.

List of Differentially Used Intronic Polyadenylation Sites during Keratinocyte Differentiation

Supplementary Data 2.

List of CPSF Core Targets from RNA-seq (4 sheets total in this file). Sheet #4: GO terms associated with the genes upregulated in CPSF CRISPRi and RNAi

Supplementary Data 3.

List of CPSF core targets with CPSF-dependent differential IpA

Supplementary Data 4.

List of HNRNPA3 Targets from RNA-seq (4 sheets total in this file). Sheet #4 -- GO terms associated with the genes upregulated with HNRNPA3 knockdown

Supplementary Data 5.

Oligos sequences used in this study
